# Eccrine Angiomatous Hamartoma With Arteriovenous Malformation: A Rare Entity Re-Explored

**DOI:** 10.7759/cureus.23669

**Published:** 2022-03-30

**Authors:** Rana S Al-Zaidi, Ghazwa Alotaibi, Mohammed Aljuaid

**Affiliations:** 1 Laboratory and Blood Bank, Anatomic Pathology Section, King Faisal Hospital, Makkah, SAU; 2 Pathology and Laboratory Medicine, King Faisal Hospital, Makkah, SAU

**Keywords:** skin diseases, malformation, arteriovenous, hamartoma, eccrine glands

## Abstract

Eccrine angiomatous hamartoma (EAH) is a rare, benign, slow-growing cutaneous lesion characterized by hamartomatous proliferation of the eccrine glands and vascular structures. It usually arises in early childhood; however, cases in adults have also been reported. It is diagnosed based on the clinical features of the lesion as well as the histopathological findings of the excised tissue. As the name indicates, EAH shows a close association with mature eccrine elements and capillary-sized blood vessels at the histopathological level. In rare instances, the vascular component can show the features of arteriovenous malformations. Here, we report a rare case of EAH with a component of arteriovenous malformation in a 39-year-old woman who presented with a foot lesion.

## Introduction

Eccrine angiomatous hamartoma (EAH) is an uncommon benign cutaneous lesion histologically characterized by peculiar hamartomatous proliferation of mature eccrine units and vascular structures in the mid and deep dermis. Typically, it presents as a solitary nodule or plaque with variable discoloration during early childhood [1]. However, cases in adults have been reported with a high incidence [2,3]. EAH is associated with other vascular lesions and tumors, including arteriovenous malformations, verrucous hemangiomas, and spindle cell hemangiomas [3]. Here, we report a rare case of EAH that showed prominent vascular proliferation with hybrid features of arteries and veins, consistent with arteriovenous malformations. This case report adds to the spectrum of histopathological findings that can be observed in this entity.

## Case presentation

A 39-year-old healthy woman presented to the general surgery clinic complaining of a non-progressing lesion on the right foot for two years. The lesion was discovered incidentally by the patient and was associated with mild pain. She had no history of hyperhidrosis, hypertrichosis, or trauma. The patient’s medical, surgical, and family history were unremarkable. On examination, a single, well-circumscribed, 1 × 1 cm firm lesion on the right foot inferior to the lateral malleolus, associated with hardening and brown discoloration of the overlying skin, was revealed. The clinical diagnosis was callus, and the lesion was surgically excised under local anesthesia.

**Figure 1:**
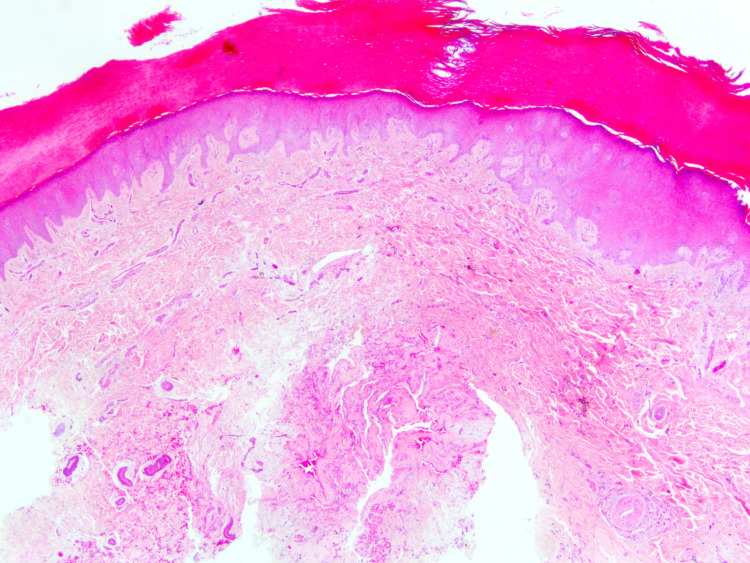
Eccrine angiomatous hamartoma showing compact orthokeratinized hyperkeratosis with mild focal acanthosis of the dermis. The lesion is apparent at the bottom of the image (hematoxylin-eosin, original magnification ×20).

**Figure 2:**
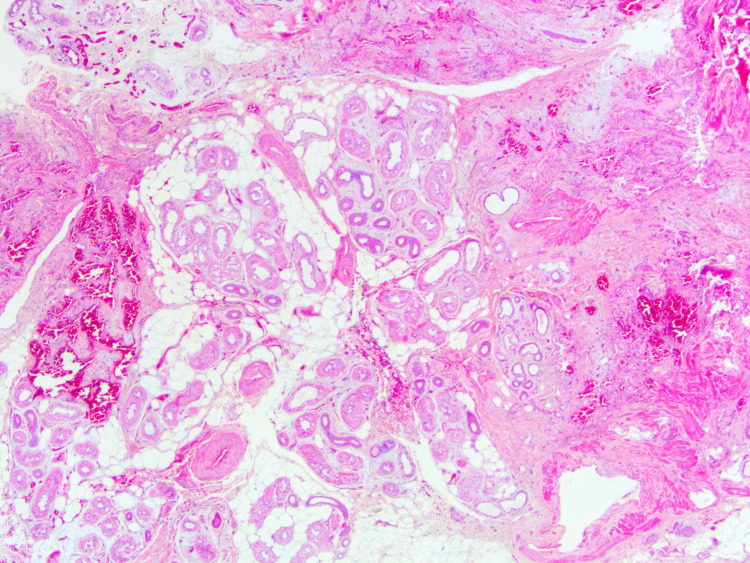
Eccrine angiomatous hamartoma showing proliferation of mature eccrine glands and ductal structures intermingled with proliferating blood vessels and mature adipose tissue (hematoxylin-eosin, original magnification ×20).

**Figure 3:**
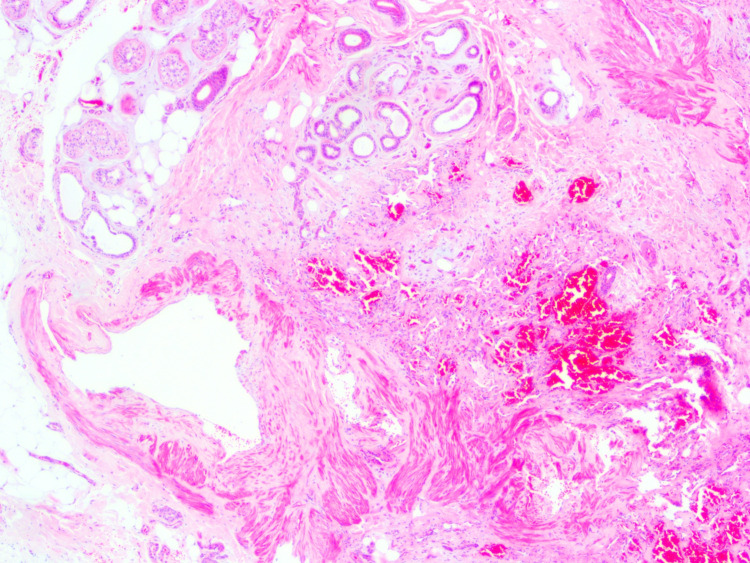
Eccrine angiomatous hamartoma showing thin-walled and focally anastomosing blood vessels. Some of the vessels are large, ectatic with distinct unevenly thickened muscular walls and partially developed internal elastic lamina (hematoxylin-eosin, original magnification ×40).

**Figure 4:**
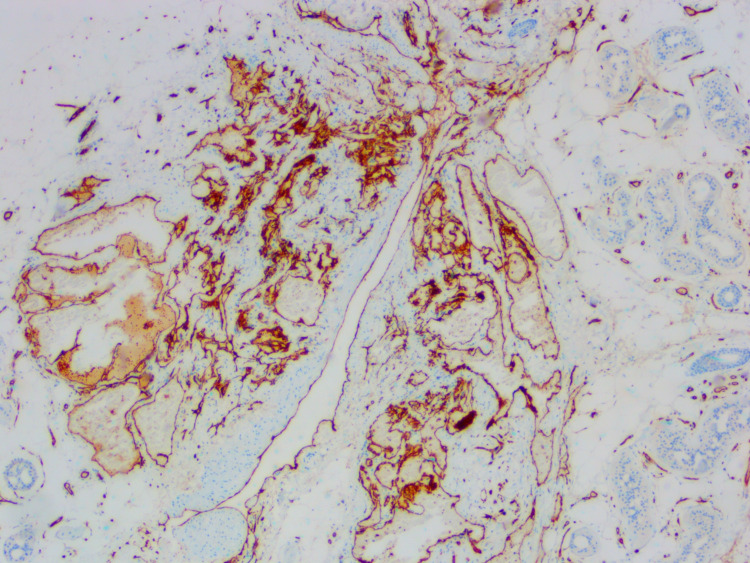
Immunohistochemical stain for CD31 highlights the endothelial cells lining the vascular component of the eccrine angiomatous hamartoma (original magnification ×40).

Grossly, the specimen consisted of an ellipse of tan unremarkable skin, measuring 2 × 0.3 × 0.2 cm. A separate irregular piece of yellow-brown soft tissue, measuring 1 × 0.5 × 0.4 cm was also identified. Histologically, the epidermis showed compacted orthokeratosis with mild focal acanthosis (Figure 1). The mid-dermis and the separate soft tissue piece showed ill-defined hamartomatous proliferation comprising lobules of mature eccrine secretory glands and ducts (Figure 2). They showed some dilation of the lumina and focal hyperplasia of the lining epithelium along with luminal tufting. The eccrine structures were closely associated and intermingled with vascular proliferation comprising lobules of small capillary-sized, thin-walled, and focally anastomosing blood vessels. Some of the vessels were ectatic, while others were large with distinct, unevenly thickened muscular walls and partially developed internal elastic lamina (Figure 3). The vessels were lined by a single layer of bland-looking endothelial cells and contained red blood cells in the lumina. A component of mature adipose tissue intermingled with eccrine lobules and vessels was also noted. The intervening stroma showed collagenous fibrous tissue with prominent myxoid changes, extravasated red blood cells, focal hemosiderin deposition, scattered mast cells, and rare small lymphocytes. There was no evidence of any cytologic atypia or increased mitotic activity within the lesion. The vascular component was highlighted using immunohistochemical staining for CD31 (clone JC70, Ready-To-Use, Ventana Medical Systems Inc., Tucson, AZ, USA) (Figure 4) and factor VIII (polyclonal, Ready-To-Use Cell Marque, California, USA). Masson trichrome staining was performed to identify the arteriovenous malformation components. Based on the histological findings, the final diagnosis was concluded as EAH with a component of arteriovenous malformation. The patient was followed up for 10 months; fortunately, she had no recurrence of the lesion.

## Discussion

EAH was first described by Lotzbeck in 1859 when he noticed an angiomatous-appearing lesion on a child’s face. The term EAH was coined by Hyman et al. in 1968 [1-3]. It is a rare, benign, cutaneous lesion that may be congenital or appear later in childhood. Cases in adults have frequently been described in the literature. EAH affects a wide age range (two months to 73 years) with no sex predilection [4]. The clinical presentation is variable, ranging from a patch to a plaque, to more commonly a nodule, or rarely a macule [1,4]. It can be skin-colored or may show yellow, brown, red, blue, or violaceous discoloration [1,3]. On rare occasions, it may show a velvety or verrucous appearance [3,5]. Its size ranges from 3 to 11 cm [3]. It typically presents as a solitary lesion; however, multiple EAH lesions have also been reported [1,3]. It can arise in any part of the body; however, it shows a predilection for the distal extremities, particularly the lower limb and palmoplantar regions. Uncommon locations include the face, neck, trunk, buttocks, sacral region, or diffusely over multiple anatomic sites. Although generally asymptomatic, EAH can be associated with mild-to-severe pain due to compression or the presence of nerve elements in the lesion [1,3]. EAH has been described as one of the 26 painful lesions of the skin [6]. Other associated symptoms that have been described include hyperhidrosis, hypertrichosis, and pruritis [1,3]. On rare occasions, a clear or yellow discharge can be observed [1]. EAH enlargement may occur in proportion to patient growth [1,3]. EAH is usually misdiagnosed clinically, probably because it is an uncommon lesion to be encountered by clinical physicians who are often unfamiliar with this entity [3]. Dermoscopic examination of EAH may show a spitzoid pattern with brown globules surrounded by pseudo-reticular depigmentation and erythematous background [7]. Another described dermoscopic appearance is a popcorn pattern, which appears as multiple yellow, confluent nodules with a popcorn shape, over an erythematous background, and linear arborizing blood vessels [5]. Based on the clinical features of EAH, the differential diagnosis includes blue rubber-bled nevus, tufted angioma, glomus tumor, vascular malformation, macular telangiectatic mastocytosis, eccrine nevus, and callus [1-3].

EAH is generally diagnosed by histopathological examination of the resected specimen. The overlying epidermis often shows none to minimal alterations, including mild hyperkeratosis, acanthosis, papillomatosis, and ulcerations. The lesion is often based in the mid or deep dermis and appears well demarcated but not encapsulated. It comprises lobules of proliferating mature eccrine ductal and glandular structures that either appear normal or exhibit hypertrophic to hyperplastic changes with dilation of the glandular lumina and, occasionally, hyperplasia of the lining epithelium with some luminal tufting or papillary fronds. The eccrine glandular structures are generally intermingled with thin-walled capillary-sized blood vessels. This is associated with variable proportions of mature adipose tissue, lymphatics, smooth muscle, pilar structures, apocrine glands, neural elements, and, rarely, bony tissue. The stroma is often loose and collagenous, with occasional cases showing myxoid changes or mucin deposition [1-4]. Often, no cytologic atypia or mitosis is seen in this lesion. Three histologic variants of EAH have been described, namely, lipomatous, follicular, and mucinous subtypes. EAH has been reported to be associated with some vascular tumors and lesions, such as arteriovenous malformation, spindle cell hemangioma, angiokeratoma, verrucous hemangioma, or overlying verrucous hemangioma-like features [3].

Our thorough literature search using the PubMed search engine revealed only two reported cases of EAH with a component of arteriovenous malformation. The first described case of this variant was in a 54-year-old man who had a red-brown nodule on his left hand that was associated with occasional pain and hyperhidrosis. Histopathological examination of the excised specimen revealed a dermal lesion with features of EAH. In addition, the proliferation of variably sized blood vessels with hybrid features of arterioles and venules was noted throughout the lesion. Of this vascular proliferation, some enlarged vessels with unevenly thickened muscular walls and partially developed internal elastic lamina were observed. Intimal proliferation with partial obliteration of the vascular lumina was also reported [4]. The constellation of these features is characteristic of arteriovenous malformations. The second described case of EAH with arteriovenous malformation was found in a retrospective study of 15 patients with EAH [3]. In their study, Lin et al. in 2012 described a 49-year-old man with a two-year history of left ankle pain and multiple cutaneous papules. The lesions were excised, and histopathological examination revealed EAH with proliferation of thin-walled vessels, accompanied by large abnormally thickened vascular structures. These morphologic findings were consistent with an arteriovenous malformation component [3]. The histologic features observed in our case were in concordance with the two previously reported cases of EAH with an arteriovenous malformation component.

The etiology of EAH is still unknown; however, congenital cases are thought to involve a defective biochemical interaction between the differentiating epithelium and subjacent mesenchyme, which leads to abnormal proliferation of adnexal and vascular structures [1,3]. In contrast, adult-onset lesions are thought to have acquired pathogenesis, as some cases arise secondary to recurrent trauma [3].

The clinical course of EAH is virtually always benign, with no reported malignant potential, and a very rare tendency for spontaneous regression [1,3,8]. The rapid growth of the lesion or increase in symptoms, particularly pain, may occur, which is thought to be due to a response to hormonal stimulation during puberty or pregnancy. Recurrence of EAH, following excision, has rarely been reported, and its underlying causes could be the regrowth of residual lesions or the development of new EAH lesions [3]. As this lesion is entirely benign, treatment is typically not required unless the patient develops severe pain that is resistant to therapy, disabling sweating, progressive enlargement, or cosmetic concerns. In general, surgical excision is the treatment of choice, ranging from excisional biopsy for small lesions to amputation of digits in the case of larger acral lesions. Non-surgical treatment options include the use of topical antiperspirants or intralesional injections of botulinum toxin in cases of hyperhidrosis. Additionally, laser therapy has been attempted but with little success [3,8,9].

## Conclusions

Our case report describes the third case of EAH with an arteriovenous malformation component, which adds to the wide spectrum of histopathological findings seen in association with this lesion. Furthermore, this finding emphasizes the hamartomatous nature of this rare cutaneous lesion.
